# Exploring L2 Listening Instruction, Self-Efficacy, and Strategy Use: A Mediation Analysis

**DOI:** 10.3389/fpsyg.2021.758757

**Published:** 2021-11-04

**Authors:** Jian Xu, Jason Fan, Kaizhou Luo

**Affiliations:** ^1^Sichuan International Studies University, Chongqing, China; ^2^Language Testing Research Centre, The University of Melbourne, Parkville, VIC, Australia; ^3^National Research Centre for Foreign Language Education, Beijing Foreign Studies University, Beijing, China

**Keywords:** L2 listening instruction, listening self-efficacy, listening strategy use, L2 listening comprehension, mediation analysis

## Abstract

This study aims to investigate different types of English listening instruction, listening self-efficacy, and listening strategy use, particularly the mediating role of self-efficacy between listening instruction and strategy use. We first examined the types of L2 instruction being employed in English as a Foreign Language (EFL) listening classrooms and then we looked into the relationships between L2 listening instruction, listening self-efficacy, and listening strategy use. The results of exploratory factor analysis demonstrated four types of English listening instruction: process-based instruction, comprehension-based instruction, self-regulation-based instruction, and strategy-based instruction. The results of structural equation modeling showed that listening self-efficacy mediated the relationship between strategy-based instruction and listening strategy use, and self-regulation-based instruction and listening strategy use. This study has implications for understanding the effectiveness of different listening teaching practices in enhancing self-efficacy and strategy use.

## Introduction

Given the vital role of English in today’s increasingly globalized world, the necessity of enhancing foreign or second language (L2) listening has received significant recognition (e.g., Field, 2019). The importance of listening itself has been considerably emphasized in English as a Foreign Language (EFL) context (Vandergrift and Goh, 2012; Goh and Vandergrift, 2021). Prior research has identified a plethora of factors which influence L2 listening comprehension, including listening task characteristics such as linguistic complexity and length of the input text (Brunfaut and Révész, 2015), task response characteristics such as complexity of the elicited response (Bloomfield et al., 2010), and listener-related factors which include listener’s metacognitive awareness (Vandergrift and Tafaghodtari, 2010), anxiety (Elkhafaifi, 2005) and motivation (Vandergrift, 2005). These factors were mainly examined from the perspective of listeners and listening input, but could have been extended to the effect of listening instruction (Siegel, 2015). The importance of teaching L2 learners how to be effective listeners cannot be overemphasized (Rubin, 1994; Turan Öztürk and Tekin, 2020). To date, however, the influence of L2 listening instruction on learners’ listening comprehension is seldom researched.

Social cognitive theory posits that individual development is affected by environmental factors, among which teachers’ instruction arguably plays a significant role in students’ self-efficacy (Bandura, 1997; Schunk, 2003). In the meantime, the self-regulated learning theory indicates that individuals’ self-efficacy level impacts their strategy use (Dörnyei, 2005). These two theoretical orientations inform the examination of the three key variables in the present study: L2 listening instruction (i.e., various teaching approaches in L2 listening), listening self-efficacy (i.e., belief in abilities to succeed in L2 listening), and listening strategy use (i.e., skills listeners employed to achieve the purpose of listening comprehension). It is hypothesized that various types of L2 listening instruction impact self-efficacy, which, in turn, affects strategy use. Regarding L2 listening instruction, L2 students are rarely taught how to listen efficiently and effectively in classroom settings (Berne, 2004). Instructors sometimes lack a well-established approach with clear pedagogical activities and effectiveness (Siegel, 2015). In addition, the consideration of listening strategy use is crucial in the sense that it can elucidate listening processes and showcase listeners’ comprehension by employing various strategies, which might be cultivated by L2 listening instruction (Siegel, 2015). The use of L2 listening strategies often distinguishes skilled listeners from those who are less skilled (Lau, 2017) and is also linked to the success in listening comprehension (Vandergrift, 2005). Among various taxonomies of listening strategies, bottom-up and top-down listening strategies play an important role in listening comprehension (Nix, 2016). In this study, bottom-up strategies refer to the processing of the listening input from the discrete units, such as word sounds, to the integral units, such as existing background knowledge, whereas top-down strategies begin with the integral units and move to the discrete units.

Moreover, self-efficacy is an essential motivational construct that refers to an individuals’ belief in their capacities to achieve certain tasks (Bandura, 1997). L2 listening self-efficacy, which denotes EFL listeners’ beliefs in their abilities to succeed or their confidence to perform well in L2 listening comprehension in this study, is a significant factor affecting listening comprehension (Rubin, 1994). We hypothesized that different types of L2 listening instruction exist in practice, and that L2 listening self-efficacy plays a mediating role between different types of L2 listening instruction and strategy use.

Previous studies mostly examined the impact of one particular listening teaching approach (e.g., Vandergrift and Tafaghodtari, 2010), yet have seldom investigated the multiple types of listening instruction that exist in contemporary L2 classrooms. Furthermore, how these different types of instruction predict students’ listening self-efficacy and strategy use remains largely unknown. To fill this gap, the present study aimed to examine the current listening instructional practices and explore which type(s) of listening instruction can improve strategy use via self-efficacy. Situated in EFL listening teaching in the Chinese tertiary context, this study investigated the types of listening instruction employed by instructors, as well as the effects of listening instructional approaches on students’ strategy use via listening self-efficacy.

## Literature Review

### L2 Listening Instruction

L2 listening instruction is a broader term which may encompass different instruction approaches (Siegel, 2014). Social cognitive theory posits that “modeling” occurs when learners acquire knowledge through the observation of models (e.g., teachers). Modeling, in this study, refers to the information that is provided by teachers about patterns, examples and sequences of behaviors that will lead to successful learning for students (Schunk, 2003). L2 listening instruction has been regarded as the “modeling” to which students attend and from which students receive and code information according to social cognitive theory and this “modeling” practice may influence an individual’s self-efficacy. In a broad sense, it is defined as the various pedagogical activities that L2 listening instructors engage in EFL teaching that aim to guide, scaffold, and improve listeners’ understanding of spoken language. In a narrow sense, it is referred to various types of listening pedagogical activities that can enhance listening self-efficacy and strategy use, which is the working definition adopted by the present study.

A review of literature reveals that a variety of L2 listening instruction types have been proposed and recommended (Siegel, 2015). Comprehension-based instruction is traditionally adopted in classrooms with a typical “listen-answer-check” pattern. It simply corrects answers to questions without analyzing the causes that underlie students’ erroneous responses (Field, 2019). Moreover, it focuses more on listening product than process, and involves an isolated and stressful learning atmosphere (Field, 2008), but this type of instruction still seems to be playing a major role in contemporary EFL classrooms (Siegel, 2014). In response to the limitations of comprehension-based instruction, a process-based approach has emerged which echoes the “skill-training principle of dividing a macro-skill into its components parts” (Field, 2008, p. 110). In most cases, a range of listening activities are employed by instructors to foster learners’ abilities in understanding and clarifying the listening process to construct meaning (Vandergrift and Goh, 2012). The difficulties of defining specific skills and demonstrating they actually exist undermine this approach in classroom teaching (Field, 2008). Limited evidence is currently available on its effectiveness (Nguyen and Abbott, 2016). Strategy-based instruction, as its name suggests, aims to equip students with listening strategies which enable them to become more competent listeners (Ngo, 2019). Of the various types of strategy-based instruction, metacognitive strategy-based instruction, which typically involves the development of listeners’ personal knowledge about listening, problem solving, directed attention, planning and evaluation, and monitoring, has been advocated by Vandergrift and Tafaghodtari (2010) and Bozorgian and Alamdari (2018) as it has been considered more effective to improve students’ listening proficiency. Drawing on a broader conception of strategy-based instruction which aims to train students how to use top-down and bottom-up strategies, Graham and Macaro (2008), Yeldham and Gruba (2014), and Yeldham (2016) have all demonstrated the effectiveness of this type of instruction. Finally, the literature suggests that L2 instructors should guide students to be autonomous and self-regulated listeners (Goh, 2000) who tend to demonstrate greater use of listening metacognitive awareness and high motivational intensity (Vandergrift, 2005). Guided reflections have often been used to direct students to evaluate their listening experiences and understand themselves as listeners (Goh, 2000). Although few empirical studies are available on the application of self-regulation-based instruction, it is vital to take this type of instruction into consideration when examining teachers’ practices in listening instruction in order to evaluate the impact of various listening instructional approaches on self-efficacy and strategy use.

Several empirical studies have been conducted to investigate L2 listening instruction. For example, Siegel (2014) found that teachers used a variety of types of L2 instruction in Japanese university classrooms, such as strategy-based instruction and comprehension-based instruction. Vandergrift and Tafaghodtari (2010) indicated the positive effect of metacognitive strategy-based instruction on improving students’ metacognitive awareness and listening comprehension. Although teachers may have some theoretical understanding about L2 listening instruction, their knowledge does not necessarily translate into effective listening teaching practices (Mendelsohn, 1995). L2 listening instructors may still persist with using theoretically unsupported practices that deviate from the core values of L2 listening instruction (Siegel, 2015). In light of this situation, identifying common practices in EFL listening classroom and how these practices predict students’ self-efficacy and strategy use is a timely contribution to the understanding and improvement of current and future L2 listening instruction (Siegel, 2014).

### Listening Self-Efficacy

Self-efficacy, a construct rooted in social cognitive theory, refers to individuals’ beliefs in their abilities and a sense of their agency to exercise control over thoughts, feelings, actions, and surrounding situations (Bandura, 1997). Self-efficacy is not dependent on one’s abilities but on what might be achieved with personal skills. Besides, this concept is associated with one’s capabilities to perform specific tasks and the levels of persistence. As far as L2 studies are concerned, a burgeoning body of empirical studies have been devoted to the psychometric properties of self-efficacy, such as in the L2 writing context (e.g., Sun et al., 2021), and the effect of self-efficacy on language learning processes and outcomes (e.g., Mills et al., 2006; Yabukoshi, 2021). Graham (2011) argued that L2 listening self-efficacy is a psychologically unobservable construct that needs a careful measurement, and that researchers should pay more attention to this notion in L2 listening, especially the impact of low self-efficacy. Listeners can hardly “rewind” to listen and pause in real-time communications and this transitory nature of processing oral input is in particular a problem for low self-efficacy listeners. Razmi et al. (2021) found that L2 listening self-efficacy was significantly correlated with L2 listening comprehension. However, empirical studies pertaining to L2 listening self-efficacy remain limited and the existent studies mainly focus on how L2 listening self-efficacy is related to L2 listening comprehension and listening anxiety. For example, research has shown that L2 listening self-efficacy is positively correlated with listening comprehension and negatively with listening anxiety (Mills et al., 2006). Few studies, however, have been conducted to explore how university students’ self-efficacy is influenced by L2 listening instruction in the context of an L2 listening course. Therefore, it is essential to explore how L2 listening self-efficacy is related to various types of L2 listening instruction.

### L2 Listening Strategy Use

The notion of self-regulated learning elaborates on the effect of self-efficacy on strategy use (Zimmerman, 2000). In addition to examining students’ views of L2 listening instruction and listening self-efficacy, it is equally important to acknowledge their listening strategy use (Fung and Macaro, 2019). Numerous studies have revealed that listening strategies are needed to improve L2 listening comprehension (e.g., Vandergrift and Tafaghodtari, 2010). Lau (2017) uncovered that skilled L2 listeners used more strategies and applied them more frequently and effectively than do their less-skilled counterparts. Bottom-up and top-down strategies are the focus of the present study because they represent two distinct psycholinguistic methods to understand oral speech and are perceived as two complementary listening processes (Graham and Macaro, 2008). In this study, we used an umbrella term, L2 listening strategy use, which encompasses both bottom-up and top-down strategies.

Empirical research has demonstrated the relationship between top-down and bottom-up strategies, both of which affect listening comprehension. Field (2008) found that any breaking down in L2 listening comprehension may be attributed to the erroneous bottom-up and top-down strategy use. Also, Nix (2016) demonstrated the interactions between top-down and bottom-up strategies, which call for the treatment of both strategies as a whole instead of two separate and independent strategies that L2 listeners employ. Given the importance of both strategies, it is necessary to investigate how they are impacted by various types of listening instruction and self-efficacy as the understanding of the influencing mechanism has implications for listening instruction.

### The Relationships Between L2 Listening Instruction, Self-Efficacy, and Strategy Use

Theoretically, L2 listening instruction influences self-efficacy, as specified in social cognitive theory (Schunk, 2003), and self-efficacy impacts listening strategy use, as signaled by the conceptualization of self-regulation. According to Zimmerman (2000), successful students not only monitor their work and persist but also judge the results of self-monitoring and thus use learning strategies more effectively. The relationship between students’ listening strategy use and L2 listening instruction has been empirically verified in the literature. For example, Chen (2009) demonstrated that the implementation of strategy instruction enabled Taiwanese students to have a greater control of their listening strategies. The findings resonate with Vandergrift and Tafaghodtari (2010) who, based on an experimental design with a sample of 106 students of L2 French, found that strategy-based instruction was effective in fostering students’ use of listening strategies. However, only one type of listening instruction was considered in this study, and it remains unclear how different types of L2 listening instruction are related to students’ strategy use. Regarding the empirical relationship between listening instruction and self-efficacy, Graham (2007, 2011) found that L2 listening self-efficacy could be boosted by listening strategy instruction, and Yeldham (2016) revealed that strategy-based instruction facilitated 33 Taiwanese non-English major freshman students’ confidence in listening and motivated them to learn how to listen. Milliner and Dimoski (2021) further discovered that strategy-focused metacognitive intervention enabled students to display a slightly more confident stance toward L2 listening. In addition, Boroumand et al. (2021) showed a positive influence of explicit teaching and utilization of concept-mapping on EFL students’ improved self-efficacy. However, these studies did not investigate whether listening self-efficacy can be enhanced by other types of listening instruction than strategy-based instruction and explicit teaching. Moreover, empirical research has also established the relationship between self-efficacy and strategy use (e.g., Wong, 2005; Rahimi and Abedi, 2014). Rahimi and Abedi (2014) survey of 371 EFL Iranian students showed that their listening self-efficacy was associated with the use of listening strategies. However, top-down and bottom-up strategies, the focus of the present study, were not examined in their study. In addition, Wong (2005) identified the positive and significant relationship between self-efficacy and general language learning strategies in an investigation of 74 pre-service teachers in Malaysia. In summary, given the limited number of studies pertaining to the relationship between listening self-efficacy and strategy use, more research in L2 listening context is still warranted.

**FIGURE 1:**
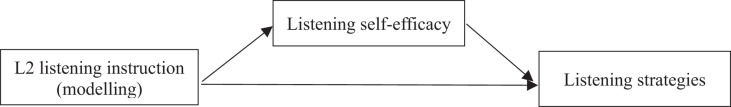
The conceptual framework portraying the relationships among listening instruction, self-efficacy, and strategy.

Amongst the prior studies examining L2 listening instruction in the EFL context, there are notable gaps yet to be addressed. First, few studies have looked into the various types of L2 listening instruction that may co-exist in EFL classrooms. Second, previous research has only examined the effect of one particular type of L2 listening instruction on self-efficacy or strategy use (e.g., Chen, 2009). Thus, it is necessary to first examine the types of L2 listening instruction that teachers use and then continue the inquiry into their effects on self-efficacy and strategy use. Third, the empirical studies reviewed above mainly focus on the relationship between L2 listening instruction and self-efficacy or strategy use, or the relationship between self-efficacy and strategy use. None of them, however, simultaneously demonstrates the relationships between L2 listening instruction, self-efficacy, and strategy use as guided by social cognitive theory and the notion of self-regulation, or clarify whether self-efficacy plays a mediating role between listening instruction and strategy use. Hence, this study seeks to go beyond merely ascertaining the impact of listening instruction on strategy use. Rather, it aims to investigate self-efficacy as a mediating variable which is influenced by instructional practices and influences strategy use, thus enabling us to uncover the mechanisms through which various types of listening instruction affect listening strategy use via listening self-efficacy. In view of previous research on the relationship between listening instruction, self-efficacy, and strategy use, the relationships between the above three variables were specified in the model below, which will be tested in this study (see Figure 1).

## The Present Study

### Research Context

This study was conducted in the context of Chinese tertiary education where English is the predominant foreign language for university students. In Chinese universities, English is a compulsory subject for most university students, who have been learning English since elementary school. A variety of English courses are offered, aimed at further improving their English proficiency. English courses are designed in accordance with the skills model, that is, listening, reading, writing, and speaking. English listening course is an integral component of the College English curriculum. The aim of the university listening course is to improve students’ English listening comprehension on a variety of topics and sociocultural issues through listening to a range of text types, such as news, documentaries, interviews, speeches, dialogues, movies. This study focuses on an English listening course as it enables us to obtain a comprehensive and nuanced understanding of listening teaching and learning practices. In the Chinese tertiary context, limited research has been conducted to explore students’ perceptions of the types of listening instruction. As such, the present study examines the different types of listening instruction in the listening classroom and their effects on listening self-efficacy and strategy use.

### Research Questions

The present study seeks to address two research questions: (1) What are the current practices of L2 listening instruction in Chinese EFL listening classes? and (2) Do L2 listening instructional practices have a significant direct and indirect relationship with students’ self-efficacy and listening strategy use? We first examined the current practices of L2 listening instruction in Chinese EFL listening classes. On the basis of the survey of current L2 listening instructional approaches, we went a step further to examine the relationships among L2 listening instruction, self-efficacy, and strategy use. The present study is significant in several aspects. First, modeling the relationships among L2 listening instruction, self-efficacy, and strategy use helps us identify the effectiveness of L2 listening instruction for enhancing self-efficacy and then strategy use in classroom contexts. Second, the present study contributes to the application of social cognitive theory to L2 listening teaching research by demonstrating the effect of teachers’ listening instruction on students’ self-efficacy; this study also sheds light on the application of self-regulation by revealing the impact of self-efficacy on strategy use.

**FIGURE 2:**
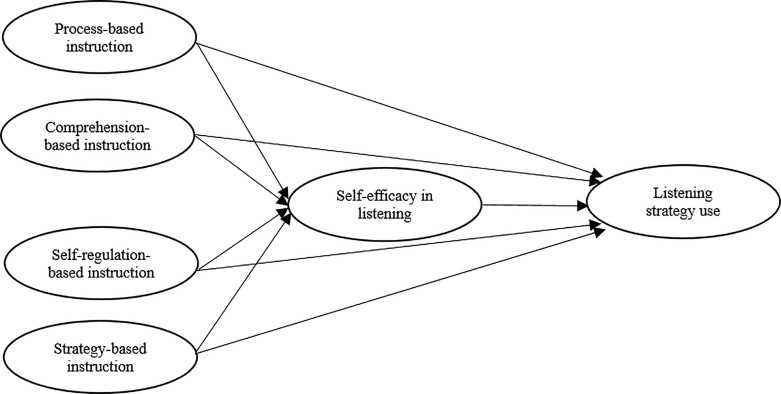
Structural equation model portraying the relationships among the variables in this study.

On this basis of literature review and theoretical frameworks, it was hypothesized that the relationships between different types of listening instruction and listening strategy use were mediated by self-efficacy in listening, as graphically illustrated in Figure 2.

## Materials and Methods

To address the two research questions, questionnaire survey was the primary data collection method in this study. In the first stage, an L2 listening instruction questionnaire (LIQ) was developed on the basis of previous research on L2 listening instruction (e.g., Siegel, 2014; Yeldham, 2016). In addition, the design of the questionnaire was informed by the authors’ experience in teaching L2 listening in the Chinese tertiary context. Next, a self-efficacy questionnaire (SEQ) was adapted based on prior studies by Zimmerman (2000) and Graham (2007, 2011). Details of the questionnaire are presented in the following measurement section. A pilot study was carried out to explore the factor structure of L2 listening instruction, self-efficacy, and listening strategy use in classrooms. Then, the revised LIQ and SEQ were distributed to another group of students along with a questionnaire that gathered information on students’ EFL top-down and bottom-up listening strategies.

### Participants

The pilot study recruited 244 first-year undergraduate students (194 Females, 50 Males) whose age ranged from 17 to 21 (*M* = 19.10, *SD* = 0.76) from a university in Beijing, China, to explore the current instructional practices in L2 listening. Employing the purposive sampling method (Lavrakas, 2008), a form of non-probability sampling method in which the members of population are chosen on purpose, four parallel classes in the same grade were chosen to participate. The first author was responsible for collecting the data while teaching the EFL listening course. The participants came from a variety of academic backgrounds, including foreign languages, finance, international relations, journalism, management and law. To explore the relationships between L2 listening instruction, self-efficacy, and strategy use, a different sample of 364 students from the same university as in the pilot study (302 Females, 62 Males) participated, aged 17 to 21 (*M* = 18.45, *SD* = 0.73). This sample drew from six different EFL listening classes where students were studying in the same year of their degree program. The data collection was completed at the end of the listening course. Participation in this study was voluntary, and participants were informed that they could withdraw from the study at any time.

### Measures

#### L2 Listening Instruction Questionnaire

Two professors in the field of English language education were invited to review the questionnaire items used in the present study and revisions were made accordingly. The LIQ was designed to measure students’ perceptions of teachers’ listening teaching practices in their English listening classes. The design of this questionnaire drew on prior reported empirical findings (Anderson, 1995; Goh, 2000; Field, 2008, 2019; Graham and Macaro, 2008; Vandergrift and Tafaghodtari, 2010; Cross, 2011; Vandergrift and Goh, 2012; Siegel, 2014; Yeldham and Gruba, 2014; Yeldham, 2016; Goh and Vandergrift, 2021). A total of 22 items representing different types of instruction were presented in Chinese. Participants were asked to judge the degree to which the description in the questionnaire items matched their perceptions of teaching on a 6-point Likert scale. In the pilot study, the Cronbach α of the scale was 0.94, and the item-total correlation ranged from 0.51 to 0.76 with the exception of one item at 0.37, indicating an overall high reliability of the LIQ. Since 0.37 was close to 0.40 which is the critical cut-off value as suggested by Ladhari (2010), it was retained in the questionnaire. Table 1 shows the revised items that were used in this study.

**TABLE 1:** Oblimin-rotated four-factor solution of LIQ.

**Item**	**Factor**			
	**1**	**2**	**3**	**4**
Different listening practices and activities helped us improve listening process in my listening class.	0.75			
My teacher organized interesting activities to teach us how to listen.	0.70			
My teacher shared and discussed personal feelings or reflections with us after listening exercise.	0.67			
Useful listening practices and activities were used to help us comprehend.	0.50			
My teacher asked us to look up the unknown words in the dictionary before listening.	0.39			
My teacher reminded us to avoid mental translation while listening.	0.37			
My teacher used quizzes or tests to improve our listening proficiency.		0.80		
My teacher used quizzes or tests to measure our listening proficiency.		0.69		
My teacher involved us in a “listening-questions-answers” pattern when teaching listening.		0.45		
My teacher asked us to listen and practice more.			−0.73	
My teacher suggested that we cultivate a good listening habit in daily life.			−0.70	
My teacher encouraged us to diagnose our own listening problems.			−0.53	
My teacher recommended useful listening websites or materials to us.			−0.40	
My teacher demonstrated us how to process information while listening.				0.76
My teacher taught us how to select different strategies based on different task types.				0.70
My teacher helped us to understand better by playing the audio sentence by sentence.				0.66
My teacher taught us specific listening strategies to improve our English listening ability.				0.65
My teacher taught us how to resolve listening problems while listening.				0.51

*Item 10 was deleted because of double loadings; item 11 was deleted because it did not load onto any of the extracted factors.*

Exploratory factor analysis (EFA), which used an extraction method of principal axis factoring and a rotation method of Oblimin with Kaiser Normalization, was first performed to estimate the factor structure of LIQ. This statistical procedure helped determine how many underlying factors could be extracted from the questionnaire items. Results showed that the eigen values of the four factors explained 64.48% of the total variance. The scree plot also confirmed that the four-factor solution was satisfactory. All the item loadings were greater than 0.30 and were significant. As displayed in Table 1, there were a total number of 18 remaining LIQ questionnaire items. We interpreted each factor according to the content of the questionnaire items.

Regarding the definition of identified types of instruction, process-based instruction (Factor 1) supports listeners in clarifying and understanding the listening materials through various activities, interactions and learning practices during the teaching process. Comprehension-based instruction (Factor 2) typically involves students listening to a text and answering related questions, after which the answers are checked by the teacher. It is a “listen-answer-check” sequence and is product-oriented in nature. Self-regulation-based instruction (Factor 3) means that students are guided in how to listen independently with the support of teachers. Finally, strategy-based instruction (Factor 4) is related to the internal cognitive processes and the teaching of listening strategies, which aims to assist students in learning how to listen. These four factors generally resonate the types of listening instruction in literature as well as in practical listening teaching practices.

#### Listening Self-Efficacy Questionnaire

The SEQ measured a single construct, i.e., students’ beliefs in their abilities to carry out listening comprehension tasks. The design of this questionnaire was informed by Graham (2007, 2011) on listening self-efficacy and by Zimmerman (2000) on the general notion of self-efficacy, and also drew from the *Motivation of Reading Questionnaire* (Wigfield, 1997) and its validated version used in the Hong Kong context (Lau, 2004). The SEQ comprises 5 items which were written in Chinese, and participants were asked to match their self-efficacy level with the questionnaire item descriptions on a 6-point Likert scale. In the preliminary study, the Cronbach α was 0.94 and the item-total correlation ranged from 0.77 to 0.90, suggesting a satisfactory reliability. The SEQ is a single-dimensional scale, which was verified by EFA results in the pilot study, with one factor explaining 81.06% of the total variance. The full scale of self-efficacy is shown in Supplementary Appendix.

#### English as a Foreign Language Listening Strategy Questionnaire

This questionnaire (ELLSQ) was derived from Nix (2016) EFL listening strategy questionnaire, which was designed and validated to assess EFL listeners’ knowledge of extant strategies. His questionnaire has two dimensions: top-down and bottom-up, which were generated by factor analysis and multi-dimensional item response theory. Nix (2016) found that the two-dimensional mode best explained participants’ listening strategy use. Specifically, bottom-up strategies (5 items) measure students’ use of individual parts to assist listening comprehension while top-down strategies (10 items) examined students’ use of integral units to assist their listening process. The English items were translated into Chinese, and then back translation was conducted which did not detect any inconsistencies. Subsequently, the Chinese version of the questionnaires was administered to participants who were asked to indicate the degree to which they used the strategies with those questionnaire statements on a 6-point Likert scale. In the pilot study, the Cronbach α was 0.93 and the item-total correlation ranged from 0.50 to 0.74, showing an acceptable reliability estimate. The EFA results from the pilot study showed a two-factor solution, which corroborated the findings of Nix (2016). However, a few problems were also identified, including: (a) item 17 had double loadings and was therefore deleted; (b) the content of item 12 was ambiguous and was also removed; and (c) item 1, which was originally classified as representing a top-down strategy, was re-classified as a bottom-up strategy. As a result, this process yielded 13 items, including 6 on bottom-up strategies (1, 2, 4, 5, 6, 7) and 7 on top-down strategies (16, 18, 19, 20, 21, 22, 23).

### Data Collection and Analysis

The revised LIQ and ELLSQ, together with the SEQ, were administered to another group of participants in this study (*N* = 302). Participants were assured of anonymity to ensure they could report their teachers’ listening instruction freely and state their own self-efficacy level and strategy use honestly and accurately. Students completed the questionnaire during their listening class.

To address the research questions, descriptive statistics, correlation analysis, reliability analysis, and item-total correlation were run in SPSS 23, and CFA was performed in AMOS 23 to examine the psychometric properties of the revised LIQ, SEQ, and ELLSQ. Next, an SEM analysis was implemented in AMOS 23 with the maximum likelihood estimation method to investigate the relationships between L2 listening instruction, self-efficacy, and listening strategy use. SEM was employed to address the complex relationships among L2 listening instruction, self-efficacy, and strategy use in this study because of its capabilities to deal with the complex relationships between multiple independent and dependent variables and to obtain explicit estimations of measurement error (In’Nami and Koizumi, 2013). A bootstrapping approach was used to examine the mediating role of self-efficacy between listening instruction and strategy use in the SEM model. In this analysis, the four types of instruction were the independent variables, listening strategy use was the dependent variable, and listening self-efficacy was the mediating variable (see Figure 2). Bootstrapping in SEM was performed to explore the mediating effect because it can generate bias-corrected confidence intervals and rule out Type II errors (Hayes, 2009). Bootstrapping is a method for data analysis and can be computed in *Mplus* software. It needs fewer inferential examinations and thus is less likely to produce Type II errors. The original item scores acted as the observed indicators for the subscales of LIQ and SEQ, and the parcel scores of bottom-up and top-down strategies were used as the two observed variables in the model. The model fit was assessed based on Kline (2011) criteria: CFI ≥ 0.9 with SRMR and RMSEA ≤ 0.08. Data screening was implemented prior to analysis to ensure the data was reliable, valid, and useable for further statistical analyses (Kline, 2011). Violations of multi-collinearity, missing data, outliers, or normality were not detected before proceeding to the SEM analysis.

## Results

### Reliability Estimates and Confirmatory Factor Analysis

Table 2 shows the internal consistency of the revised LIQ, ELLSQ, and SEQ. All the subscales demonstrated a high internal consistency except for the subscale of comprehension-based instruction, which showed a moderate reliability. The findings of the reliability estimates revealed that the reliability of all the questionnaires used was acceptable. In addition to reliability estimates, CFA was performed on all three questionnaires. Items were specified on the latent variables in a measurement model and the degree to which the data fit the model was calculated. The model-fit result of the CFA of LIQ with four factors was acceptable (χ^2^/df = 3.54, *p* < 0.00, CFI = 0.90, SRMR = 0.07, RMSEA = 0.08). The single-dimensional construct of SEQ also yielded a satisfactory model fit (χ^2^/df = 1.37, *p* < 0.00, CFI = 0.99, SRMR = 0.01, RMSEA = 0.03) and the CFA of two-factor solution of ELLSQ generated a satisfactory model fit (χ^2^/df = 4.11, *p* < 0.00, CFI = 0.92, SRMR = 0.05, RMSEA = 0.09). The factor loadings of most items on the specified latent variable were high, indicating that those items defined the latent variables accurately^1^.

**TABLE 2:** Reliability estimates of the questionnaires in the main study.

**Subscale**	**No. of items**	**Cronbach α**	**Mean**	***SD***
LIQ: Process-based instruction	6	0.82	4.54	0.79
LIQ: Comprehension-based instruction	3	0.60	4.97	0.76
LIQ: Self-regulation-based instruction	4	0.80	5.23	0.69
LIQ: Strategy-based instruction	5	0.86	4.47	0.93
SEQ: Self-efficacy	5	0.94	3.38	1.24
ELLSQ: Bottom-up strategies	6	0.87	4.71	0.74
ELLSQ: Top-down strategies	7	0.87	4.30	0.85

### Descriptive Statistics and Correlation Analysis

The results of descriptive analysis of the subscale of the LIQ, SEQ, and ELLSQ are presented in Table 2. As indicated in this table, the means of process-based and strategy-based instruction were lower than those of comprehension-based and self-regulation-based instruction. Among the four dimensions of LIQ, the mean of self-regulation-based instruction was the highest. Notably, the mean score of self-efficacy was slightly below 3.5. In addition, the mean score for the perceived use of bottom-up strategies was higher than that of the top-down strategies, both of which were higher than the middle point (3.5). Table 3 describes the bivariate correlations among all the latent variables in the three questionnaires, namely process-based instruction, comprehension-based instruction, self-regulation-based instruction, strategy-based instruction, self-efficacy in listening, top-down strategies, and bottom-up strategies. The correlation results showed that the four types of L2 listening instruction were all significantly associated with bottom-up and top-down strategies; the four types of instructional practices were all significantly related to self-efficacy which also correlated significantly with bottom-up and top-down strategies.

**TABLE 3:** Zero-order bivariate correlations among the variables.

	**1**	**2**	**3**	**4**	**5**	**6**	**7**
Process-based instruction	1						
Comprehension-based instruction	0.39**	1					
Self-regulation-based instruction	0.49**	0.29**	1				
Strategy-based instruction	0.75**	0.32**	0.42**	1			
Self-efficacy	0.30**	0.22**	0.04**	0.33**	1		
Bottom-up strategies	0.43**	0.30**	0.35**	0.42**	0.46**	1	
Top-down strategies	0.47**	0.25**	0.25**	0.45**	0.62**	0.65**	1

*^∗∗^p < 0.01 (two-tailed).*

### Relationships Between L2 Listening Instruction, Self-Efficacy, and Strategy Use

**FIGURE 3:**
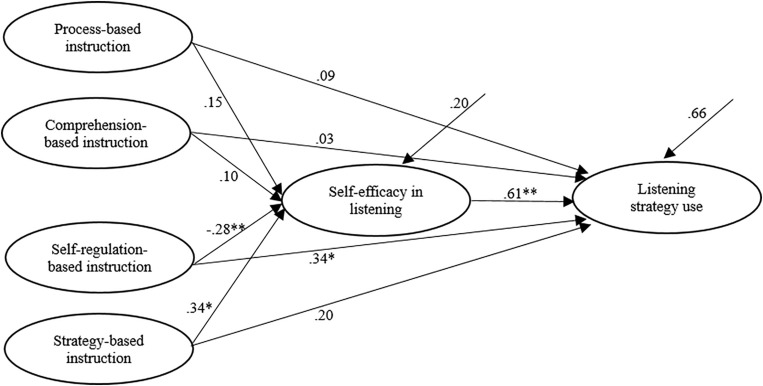
Structural equation model describing the relationships among variables measured in this study. *p < 0.05, **p < 0.01.

Results showed that the model fit of the SEM mode was satisfactory (χ^2^/d*f* = 3.10, *p* < 0.00, CFI = 0.90 SRMR = 0.07, RMSEA = 0.07), suggesting that the questionnaire data fit the hypothesized model well (see Figure 3).

The four types of L2 listening instruction were correlated with each other and are presented in Table 3. In the interest of clarity, the inter-correlation arrows between four types of listening instruction were not shown in Figure 3. Without considering the mediator of self-efficacy, results of the total effect showed that only strategy-based instruction was significantly associated with listening strategy use (β = 0.40, *p* < 0.05). Regarding the direct effects as shown in Figure 3, self-regulation-based instruction was significantly but negatively related to self-efficacy (β = −0.28, *p* < 0.01), whereas strategy-based instruction was correlated with self-efficacy significantly and positively (β = 0.34, *p* < 0.05); self-efficacy in listening was also significantly and positively associated with listening strategy use (β = 0.61, *p* < 0.01). When it comes to the mediating effect (indirect effect) of self-efficacy, the procedures of mediation were conducted following the guidelines of Zhao et al. (2010). The mediating effect was then checked by examining the significance of the indirect effect. If there was no zero between the lower and higher limits, it meant that the indirect effect was significant and there was indeed a mediating effect (Zhao et al., 2010). The results of the indirect effect of the four types of listening instruction on listening strategy via the mediator of self-efficacy showed that self-efficacy mediated the relationship between self-regulation-based instruction and listening strategy use because there was no zero between −0.27 and −0.08; similarly, self-efficacy was found to mediate the relationship between strategy-based instruction and listening strategy use, as there was no zero between 0.04 and 0.37, either (see Table 4). On the other hand, self-efficacy did not mediate the relationship between process-based instruction and listening strategy use; nor did it mediate between comprehension-based instruction and listening strategy use, for the indirect effect of these variables was insignificant. However, it should be noted that the results showed that the indirect effects of self-regulation-based instruction on listening strategy use were negative. That is, self-regulated instruction negatively influenced self-efficacy which then positively influenced students’ strategy use.

**TABLE 4:** The significance of the mediating effect of self-efficacy between listening instruction and strategy use.

**Dependent variable**	**Independent variable**	**Bootstrapping**		
		**Indirect effect**		
		**BC 95% CI**		**Estimate**
		**Lower**	**Upper**	
Listening strategy use	Process-based instruction	–0.11	0.30	0.09
	Comprehension-based instruction	–0.04	0.18	0.06
	Self-regulation-based instruction	−**0.27**	−**0.08**	−**0.17**
	Strategy-based instruction	**0.04**	**0.37**	**0.20**

Finally, the squared multiple regression for self-efficacy in listening was 0.20, implying that 20% of the variance of listening self-efficacy was jointly explained by process-based instruction, comprehension-based instruction, self-regulation-based instruction, and strategy-based instruction. The squared multiple regression for listening strategy use was 0.66, indicating that 66% of the variance of listening strategy use was jointly accounted for by these four instruction types and listening self-efficacy.

## Discussion

This study explored the current L2 listening teaching practices at the university level and examined the relationships among students’ perceived L2 listening instruction, self-efficacy in listening, and listening strategy use. It was hypothesized that the four types of listening instruction, identified by EFA and consistent with existing literature, enhanced students’ self-efficacy, thereby increasing their use of listening strategies. However, the relationships between listening strategy use and both process-based instruction and comprehension-based instruction were not significantly mediated by listening self-efficacy. This might be explained by the fact that comprehension-based instruction is such a traditional teaching approach that the teacher-centered, single “listening-questions-answers” largely fails to encourage student improvement systematically (Siegel, 2015). Similarly, the various learning activities and emphasis on processes in process-based instruction may not meet students’ needs concerning learning “how to listen” and the effectiveness of this type of instruction may need to be further explored (Nguyen and Abbott, 2016). It was not surprising to find that strategy-based instruction first increases students’ self-efficacy and then improves their strategy use, which corroborates previous findings (e.g., Graham, 2007, 2011; Graham and Macaro, 2008; Vandergrift and Tafaghodtari, 2010). Self-efficacy in listening was also found to mediate the relationship between self-regulation-based instruction and strategy use, which partially resonates with Vandergrift (2005) finding that self-regulated listeners’ learning orientation enhances their listening strategy use.

However, it is also interesting to note that self-regulation-based instruction has a negative indirect effect on strategy use. Specifically, self-regulation-based instruction has a negative direct effect on self-efficacy which has a positive effect on listening strategy use. Although encouraging students to be self-regulated learners has long been advocated in educational settings (Zimmerman, 2000), it seems that teachers’ motivation styles, models, or encouragements are possibly ineffective in boosting self-efficacy and may even cause students’ self-efficacy to decline in some L2 listening settings. This could be caused by the nature of L2 listening comprehension, which is complex, dynamic, and transient (Rost, 2011). These characteristics are likely to cause listening difficulties for listeners (Rost, 2011), and discourage them from becoming self-regulated listeners (Goh, 2010), thus partly explaining the negative correlation between self-regulation-based instruction and listening self-efficacy identified in this study.

As shown in the descriptive analysis (see Table 2), the mean of self-regulated-instruction was the highest which signifies the highest frequencies as reported by students, followed by process-based and comprehension-based instruction, both of which are characterized by teachers’ dominance in the classroom with limited opportunities for students to be involved in the learning process. Those three types of listening instruction were found to coexist in listening teaching practices. Hence, the effect of self-regulation-based instruction on students’ self-efficacy and strategy use may be affected by the overlap with process-based and comprehension-based instruction. As such, it is advised that in EFL listening classrooms, rather than simply asking students to be self-regulated listeners, instructors should offer tools to support and direct students to be self-regulated listeners. Those tools could be study plans that offer a to-do list with different listening tasks and listening materials that arouse students’ intrinsic interest; they should guide students to learn how to take notes, and provide emotional support where necessary to maximize the effects of listening instruction. In other words, the self-regulation-based instruction should be assisted by teachers’ supportive practices to maximize its benefits to students. In Chinese society, learning simply to pass examinations and compete for success is a deeply rooted practice (Cheng, 2008). Students may not fully appreciate self-regulated-based instruction as they may not believe learning by themselves will enable them to get a higher score, and they tend to rely on their teachers. Instructors, thus, should position themselves to convince students of the effectiveness of self-regulated instruction. This explains why strategy-based instruction whose aim is to teach students how to listen, can so effectively improve their self-efficacy and strategy use in Chinese EFL context.

Based on the research findings of the present study, there is a need to transform from a teacher-dominated approach (e.g., comprehension-based and process-based instruction) to a student-centered approach, such as the strategy-based approach, that enables students to play an active role in listening class instead of passively receiving listening input. The strategy-based instruction, to some degree, emphasizes the role of students and guides them how to listen, so it is more effective for the improvement of self-efficacy and strategy use. However, it does not mean that instructors are no longer important in listening classes. Instead, the instructor’s role is to guide, scaffold, and facilitate students’ listening learning rather than simply asking students to listen on their own (Goh, 2010). The reason why students feel self-efficacious under the strategy-based instruction might be that they obtain the ways of how to listen and actively engage in listening comprehension under the guidance of instructors in a more student-centered classroom. However, it remains unclear how long students’ knowledge of “how to listen” could sustain, which can be investigated in future research.

To enhance students’ listening strategy use is meaningful and significant in L2 listening classrooms as research has shown that the effective use of listening strategy use will positively predict listening comprehension (Vandergrift, 2005). In addition, according to our research findings, the increase of students’ listening strategy use does not necessarily result from a direct strategy-based instruction. Rather, the influencing path from strategy-based instruction to listening strategy use is mediated by listening self-efficacy, thus highlighting the role of listening self-efficacy. Students could build self-efficacy through strategy-based listening instruction, as suggested by our study. It can be safely concluded that students would boost self-efficacy through their own mastery experiences (Bandura, 1997) under the guidance of instructors. It is students themselves that strive to improve listening self-efficacy and strategy use under instructors’ guidance, so it is important for them to persist when facing challenges and then to achieve the learning goals despite experiencing failure during the learning process.

Informed by social cognitive theory and the notion of self-regulation, this study proposed a hypothesized model which was partly supported and validated by the data that we collected. That is, teachers’ listening instruction is shown to influence students’ listening self-efficacy, which, in turn, impacts their listening strategy use. Moreover, it also contributes to the evidence that not all teacher modeling is effective in enhancing students’ self-efficacy and strategy use. Despite teachers’ intentions to influence students positively, their perceptions of or attitudes toward these modeling matters should not be ignored. Therefore, a qualified instructor may realize that they can boost students’ self-efficacy not only through modeling but also by acting as a trusted voice of encouragement and someone who demonstrates flexibility in L2 listening instruction, satisfies students’ needs, and helps them develop learning skills and recognize the ways in which they can demonstrate competence and step into the ring.

The findings of this study have several implications for EFL listening instruction. First, the higher average scores of subscales of instructional practices suggest that L2 listening instructors have generally adopted a variety of approaches to teaching English listening in their classes, but it is necessary for teachers to be aware of the effectiveness of different instructional approaches. Classroom instructors should not give equal weight to these four types of instruction. Rather, they should shift their focus from comprehension-based and process-based instruction to strategy-based instruction, which enables students to develop a wider range of listening skills and strategies that can be extrapolated beyond the classroom (Lynch, 2009). Even if instructors prefer to incorporate some elements of a traditional approach, it is sensible to pay more attention to strategy-based instruction. Second, encouraging students to be self-regulated learners should be widely advocated, as proposed by the Zimmerman (2000), but what matters most is how instructors guide students to be self-regulated. The negative correlation between self-regulation-based instruction and students’ self-efficacy in this study calls for greater support from instructors for students’ self-regulated learning during and after listening class. Instructors also need to be mindful of the ways they deliver feedback to avoid negative impacts on students’ self-efficacy. Third, process-based instruction needs further refinement, improvement, and research. The findings of this study suggest that process-based instruction does not boost students’ self-efficacy and strategy use. According to the literature, this type of instruction can at least offer scaffolding for listeners (Siegel, 2015). However, a consensus on its effectiveness has not been reached, which implies that teachers should not abandon such an approach but should perhaps instead consider how to refine it and better apply the theories of process-based instruction to their classes. For example, L2 listening instructors could adjust the teaching sequences and organize activities more systematically to achieve the goals of process-based instruction. Finally, it is anticipated that the classroom teaching could ultimately enable students to communicate effective in real-time communications. Perhaps it is necessary for L2 instructors to create a natural listening-speaking environment by using some authentic listening tasks to extend L2 listening teaching to communications in the real world. Based on those authentic tasks, instructors may help students learn how to listen, which would in turn maximize the effectiveness of L2 listening instruction in classroom settings.

## Conclusion

This study shows that listening self-efficacy mediates the relationship between strategy-based instruction and listening strategy use, and that between self-regulation-based instruction and listening strategy use. Despite the implications for listening instruction, we acknowledge a few limitations with this study. First, the findings may not be generalizable to L2 learners in other contexts. In addition, the present study is cross-sectional and therefore unable to reveal the developmental relationships between the variables of interest which could be identified in a longitudinal SEM model. A future longitudinal study could collect data on multiple time points to clarify the developmental relationships among the variables. Finally, the investigation into the types of L2 listening instruction could be significantly enriched by having multiple data sources, such as interviews, classroom observations, and students’ reflections. Through modeling the relationships between listening instruction, self-efficacy and strategy use, we hope that this study has contributed to the understanding of L2 listening instruction and its influence on students’ listening self-efficacy and strategy use in EFL contexts.

## Data Availability Statement

The raw data supporting the conclusions of this article will be made available by the authors, upon reasonable request.

## Ethics Statement

The studies involving human participants were reviewed and approved by the Sichuan International Studies University. The participants provided their written informed consent to participate in this study.

## Author Contributions

JX collected the data and wrote the manuscript. JF revised the manuscript. KL wrote and revised the manuscript. All authors contributed to the article and approved the submitted version.

## Conflict of Interest

The authors declare that the research was conducted in the absence of any commercial or financial relationships that could be construed as a potential conflict of interest.

## Publisher’s Note

All claims expressed in this article are solely those of the authors and do not necessarily represent those of their affiliated organizations, or those of the publisher, the editors and the reviewers. Any product that may be evaluated in this article, or claim that may be made by its manufacturer, is not guaranteed or endorsed by the publisher.

## Footnotes

1 In the interest of conciseness, those factor loadings are not displayed here.
