# Supplementary material for: Exploring L2 Listening Instruction, Self-Efficacy, and Strategy Use: A Mediation Analysis
Source: Front Psychol. 2021 Nov 4;12:758757. doi: 10.3389/fpsyg.2021.758757 (PMC8599935; doi:10.3389/fpsyg.2021.758757)
Supplement: Supplementary file 1 [file Table_1.docx]

**Appendix**

Scale of self-efficacy in listening

| 1. I feel confident about my English listening proficiency. |
| --- |
| 1. I feel confident when listening to English. |
| 1. I believe that I can communicate with foreigners or other people in English very well. |
| 1. I think that I can achieve good test scores on important English language tests. |
| 1. English listening learning is easy for me. |
